# Nano-Mediated Photodynamic Therapy for Cancer: Enhancement of Cancer Specificity and Therapeutic Effects

**DOI:** 10.3390/nano8110923

**Published:** 2018-11-08

**Authors:** Ivan Mfouo Tynga, Heidi Abrahamse

**Affiliations:** Laser Research Centre, Faculty of Health Sciences, University of Johannesburg, P.O. Box 17011, Doornfontein 2028, South Africa; ivant@uj.ac.za

**Keywords:** cancer, cancer therapies, cancer recurrence, stem cells, porphine-related macrocycles, enhanced targeting, nanomedicine

## Abstract

Deregulation of cell growth and development lead to cancer, a severe condition that claims millions of lives worldwide. Targeted or selective approaches used during cancer treatment determine the efficacy and outcome of the therapy. In order to enhance specificity and targeting and obtain better treatment options for cancer, novel modalities are currently under development. Photodynamic therapy has the potential to eradicate cancer, and combination therapy would yield even greater outcomes. Nanomedicine-aided cancer therapy shows enhanced specificity for cancer cells and minimal side-effects coupled with effective cancer destruction both in vitro and in vivo. Nanocarriers used in drug-delivery systems are very capable of penetrating the cancer stem cell niche, simultaneously killing cancer cells and eradicating drug-resistant cancer stem cells, yielding therapeutic efficiency of up to 100-fold against drug-resistant cancer in comparison with free drugs. Safety precautions should be considered when using nano-mediated therapy as the effects of extended exposure to biological environments are still to be determined.

## 1. Introduction

Cancer is a major cause of death worldwide, with a projected increase to 19.3 million new cases in the year 2025 with the most vulnerable being the low- and middle-income populations [1]. In normal scenarios, the regulation of both cell growth and development signals operates well and permits the induction of tolerance, resistance, or cell death response after exposure to both external and internal stimuli [2]. Defective regulation leads to excessive cell growth, abnormal apoptosis, carcinogenesis, and eventually tumor metastasis and cancer. Defects in the mechanisms of apoptosis are primordial for tumor initiation, tumor proliferation, and metastatic progression [3]. Tumor or cancer cells have the capability to undergo cell survival beyond the normal life expectancy and atypically multiply with a higher level of cell proliferation than normal cells. Most current cancer therapies are good at targeting neoplastic cells and affecting fast-proliferating cells, including normal cells of skin, hair, and gastrointestinal cells. The search for novel and targeted-cancer therapies is justified by the inability of current treatments to effectively cure cancer without damaging normal cells [4]. Other cancer therapies are effective in eradicating cancer but only partly or temporally before tumor recurrence [5]. Tumor recurrence is predictable in most cases, owing to acquired drug-resistance or the development of cancer stem cells [6].

Cancer recurrence can be a useful tool to assess the suitability of cancer burden and treatment. Nowadays, large numbers of cancer deaths occur as a result of cancer recurrence in patients who had previously completed treatment programs and thereafter appeared to be disease-free [7]. Cancer recurrence is the reappearance and manifestation of cancer after a period known as remission, a post-treatment and disease-free period [8]. Usually, recurrent cancers are worse and thought to be more aggressive than their previous states. The fact that there is no precise duration of remission, shorter periods make it difficult to differentiate recurrence from cancer progression. The fear of cancer recurrence is an uprising concern among patients and can be predicted by a thorough analysis of certain factors [8,9]. Cancer recurs as the result of some cancer cells that remain unaffected after treatment and later become detrimental, causing either local, regional, or distant symptoms. Therefore, proper cancer selectivity or targeting approaches stand as essential criteria for any cancer therapy [10]. Inappropriate drug-targeting is one of the most common causes of cancer drug resistance to conventional treatments [11]. Others mechanisms of direct or indirect drug resistance in human cancer may include drug efflux, drug inactivation, DNA damage repair, cell death inhibition, epigenetics, and epithelial-mesenchymal transition. Any or a combination of these mechanisms render unwanted cells capable of tolerating therapeutic agents, developing drug tolerance or resistance, which can be graded as disease-specific or evolutionarily conserved (microorganisms and humans) [11,12]. The search for effective treatment modalities is encouraged with treatment combination allegedly more effective than taking any treatments separately. Such combinations are thought to be able to counteract intrinsic or acquired drug resistance of cancer cells [13,14,15].

**Figure 1:**
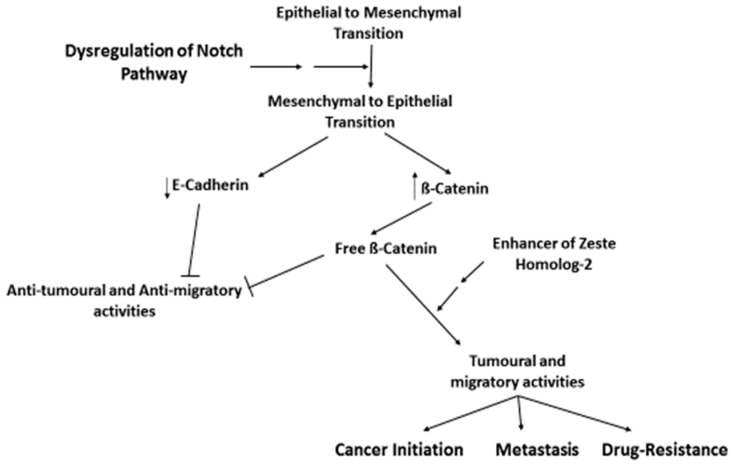
The role of stem cells in carcinogenesis, cancer metastasis, and drug-resistance. The dysregulation of the Notch favors the mesenchymal to epithelial transition of stem cells over the epithelial to mesenchymal one, leading to the decrease of E-cadherin and increase of free beta-catenin, and subsequent stimulation of tumoral and migratory functions, which can be mediated by certain proteins, such as enhancer of zeste homologue-2 protein.

According to the stem cell theory of cancer, a marginal and undifferentiated side-population of cells known as cancer stem cells (CSCs) or tumor initiation cells (TICs), possessing stem-like properties, could be held responsible for initiating, propagating, and sustaining cancer [16]. On top of the characteristic features of normal stem cells, CSCs possess tumorigenic phenotypes such as multidrug resistance, uncontrolled growth and proliferation, tissue invasion, epithelial-mesenchymal plasticity, and high levels of expression of anti-apoptotic proteins and drug efflux pumps [17,18]. They are capable of renewing themselves, differentiating into other cells, spending extended time in the non-dividing G0 cell cycle stage, and exhibiting altered phenotypes. Such modifications confer to CSC the ability to differentiate from common cancer cells, to overexpress certain drug efflux transporters, and to develop multiple drug resistance, cancer recurrence, and metastasis [19]. A correlation has been established between epithelial-mesenchymal transition (EMT)-type cells, CSC, and microRNAs (miRNAs). A miRNA is a short and non-coding RNA molecule that can play a crucial role in RNA silencing and post-transcriptional regulation [20]. EMT cells possess CSC-like characteristics, and CSC displays a mesenchymal phenotype. Both the formation of CSC and the acquisition of an EMT phenotype are connected by the expression of aberrant miRNAs. Effective cancer treatments targeting miRNAs should be able to affect the regulation of EMT that could cause the suppression of CSC or EMT-type cells, as miRNAs appeared to be the essence of the cause of drug resistance and cancer recurrence, see Figure 1.

## 2. Cancer Therapy: Photodynamic Therapy as a Solution for Cancer Relapse

### 2.1. Conventional Cancer Therapies

Surgical resection and chemotherapy are the most common conventional cancer treatments and cure less than 50% of all patients with cancer [21]. Surgical resection is the most effective treatment with almost 45% of cases cured after the entire or partial removal of affected organs [22]. When not removed, the exposure of malfunctioning organs to chemotherapeutic agents, causes damage to rapidly proliferating cells, both neoplastic cells as well as normal cells in the bone marrow, macrophage, digestive tract, and hair follicles [23,24]. The degree of the side effects enforces the necessity for modification of treatment parameters, such as changes in dosage, in time intervals between repeats, or simply discontinuing the chemotherapeutic program due to the low survival rates after therapy [23,25,26]. Numerous side effects, nonspecific targeting, and poor delivery of anticancer agents are motives for novel and effective cancer therapy modalities, which aim to identify and treat solely cancer cells. 

### 2.2. Photodynamic Therapy

**Figure 2:**
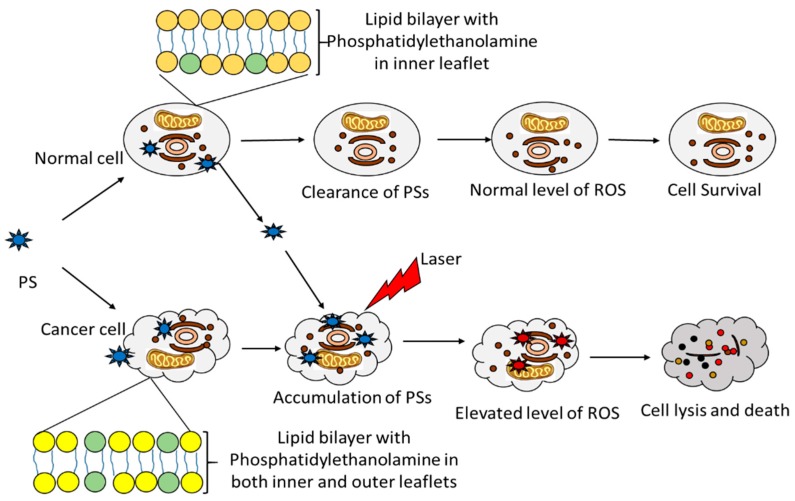
One of the processes by which photosensitizer (PS) molecules accumulate in cells with phosphatidylethanolamine in both inner and outer leaflets of the lipid bilayer membranes, like in cancer cells. Laser irradiation activates PS and reactions, leading to the generation of reactive oxygen species (ROS) and cell destruction.

Photodynamic therapy (PDT) is a treatment approach suitable for certain types of conditions which are not cured by surgical resection and include various cancer types. PDT is considered as a promising treatment modality, presenting significant effectiveness and limited side effects, while numerous side effects are associated with chemotherapy [27,28]. In PDT, a non-toxic agent known as photosensitizer (PS) is administered and taken up by tumor cells, before being activated by light of a specific wavelength that matches its absorption properties. Then, light-activated PS induces selective damage to tumors and surrounding vasculature in the presence of molecular oxygen, see Figure 2 [28]. The success of PDT solely depends on the choice of PSs, characterized by chemical purity, activation with a wavelength appropriate for tissues (near the infrared region), selectivity to neoplastic cells, and development of a long-lived triplet excited state. Some of the good examples of PSs used in PDT are methylene blue, hematoporphyrin, chlorins, photodithazine, curcumin, phthalocyanines, and hypericin [28,29,30]. Type I or type II reactions can result from an effective activation of PS in the presence of molecular oxygen. Type I reaction is related to a low oxygen level in treated tissues (neoplastic), and depends on the interaction between the triplet excited state of PS (3PS*) and the treated tissues, which act as a substrate for 3PS*. This interaction generates radicals, which are able to interact with oxygen to produce reactive oxygen species (ROS). Generated ROS cause damage to the treated tissues and, subsequently, result in cell death [31,32]. Type II reaction is related to a high level of oxygen in treated tissues (commonly human neoplastic), and depends on the direct interaction between the triplet excited state of PS (3PS*) and oxygen to generate a singlet excited state of oxygen. The latter is a highly reactive and toxic molecule, able to readily damage treated tissues and cause subsequent cell death [31,33,34]. The induced cell death response is dependent on the subcellular localization of PS. A mitochondria-localized PS would likely induce an apoptotic response whereas PS that localized in lysosomes induces an apoptotic response via cleavage of BID and/or a necrotic response after a supra-lethal PDT dose [35]. In order to enhance their therapeutic efficiency, PSs are constantly being developed and improved by conjugating them with other molecules. Previously, PDT was restricted to superficial conditions due to the inaccessibility of light into deeper areas. Recent developments indicated that PDT would now be suitable for both conditions, including brain and liver cancers, by means of a novel wireless device that activate PSs [36].

### 2.3. Tetrapyrrolic Photosensitizers

**Figure 3:**
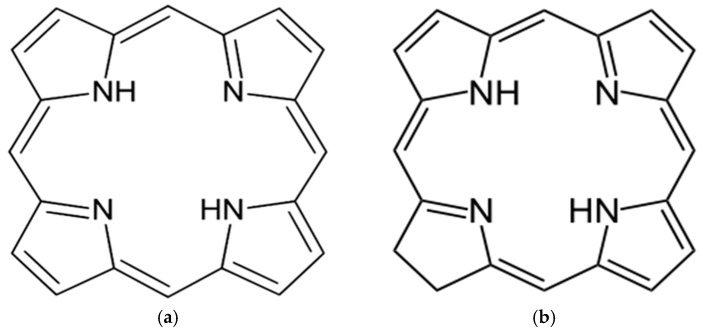
(**a**) Structure of porphine, a macrocyclic ring consisting of four pyrrole-like rings; (**b**) structure of chlorin, a macrocyclic ring consisting of three pyrrole-like rings, both joined by four methine groups. Pyrrole is a heterocyclic aromatic organic compound with a five-membered ring structure.

Among the most commonly used PSs for PDT, those with tetrapyrrolic structures are particularly interesting. They are natural pigmented molecules with four pyrrole rings, joined by carbon-carbon interactions, and both linear and cyclic tetrapyrroles may be found. The linear form may or may not undergo configurational changes such as stereochemical, homosequential, or stereohomosequential changes and/or Fischer-Rosanoff conventions to give rise to their cyclic counterparts [37]. Porphyrins have important biochemical properties and are derived from porphine, an organic aromatic and heterocyclic compound, consisting of four pyrrole rings, which is an essential ring system, see Figure 3a [38,39]. Another compound with a central aromatic ring structure is chlorin, which contains at its core three pyrroles and displays no aromaticity throughout its perimeter, see Figure 3b [40].

Obtained through the cyclotetramerization of phthalic acid derivatives and phthalimides, phthalocyanines are structurally similar to porphyrins and porphyrazines [41]. This family of tetrapyrrolic structures with incorporated central metal ions is also used for the treatment of non-invasive cancers, and designated as a second generation of PSs with improved photophysical and photodynamic properties over those of porphyrins. These tetrapyrrolic macrocycles strongly absorb light in the near-infrared region of the visible spectrum and are proven to effectively kill malignant tumors during PDT [42,43,44]. One of the major downfalls of this family of PSs is the lack of adequate water solubility. This issue can be overcome by adding charged particles, carbohydrates, or peptide ligands to the periphery of phthalocyanines to give rise to third generation PSs [44].

### 2.4. Enhanced Targerting Approach

As discussed earlier, miRNAs play essential roles in cancer development as well as regulatory roles by targeting mRNA for cleavage or translational repression. The increased expression level of miRNA-210, miRNA-296, and induced apoptosis related-miRNA were demonstrated after PDT-mediated treatment of Hela cells. PDT-prompted hypoxia led to a subsequent increased expression of miRNA-210 as well as vascular endothelial growth factor (VEGF). The miRNA-210 is a predominant miRNA activated under hypoxic environments. VEGF stimulated the expression of miRNA-296 and restored blood supply to cells that have become deprived of oxygenated blood [45,46]. Thus, PDT causes damage to the tumor microenvironment, which is both a cause and consequence of tumorigenesis. An effective cancer treatment should be able to stimulate biological processes such as ROS generation, hypoxia, and VEGF regulation of angiogenesis to target the tumor microenvironment and prevent cancer [47]. The efficiency of PDT was established after assessing its impacts on the miRNome with the overexpression of phototoxic-miRNAs 130a, 93, 25, or inhibition of resistive-miRNAs 20a, 141, 200a, 200c, and 203, which all improved the vulnerability of insensitive cell lines (drug-resistant) towards PDT [27].

## 3. Nanomedicine for Better Cancer Therapy

Nanotechnology is a multidisciplinary field and has emerged from the junction of chemistry, biology, applied physics, optics, digital analysis, and materials science. This evolving and interdisciplinary field mainly involves the design, characterization, manufacture, manipulation, and application of structures at the nanometer scale (nano: one billionth). Such structures are known as nanoparticles and are of particular interest as seen by the growing popularity and publications on nanoparticles or nanomaterials [48,49,50]. These can be attributed to their physicochemical features, among which are their rigidity, hydrophobicity, size, and charge, which portray this technology as tremendously potent in solving societal issues including health-related ones [51]. When used to monitor, repair, and regulate molecular activities of human biological systems, it is known as nanomedicine; the specialized application of nanotechnology to achieve a reliable diagnosis as well as effective therapy. It includes aspects such as nanoparticle drug delivery, which currently constitutes an extensively studied area and shows potentialities for both molecular nanotechnology and nano-vaccinology [52,53,54,55]. The increased attractiveness of nanoparticles mediated-therapy rests on the abilities of nanomaterials to deliver hydrophobic-like treatment to diseased areas by overcoming biological barriers [56]. Noble metal nanoparticles comprise gold, silver, and platinum nanoparticles and are of particular interest in providing such treatments. They exhibit localized surface plasmon resonance due to their strong optical absorption and scattering propensities. They can readily interact with biomolecules equally at the membrane surface and inside cells. Such nanoparticles can enhance specific signals during diagnosis and are able to effectively treat critical illness such as bony and dental conditions, various cancers, diverse infections, tuberculosis, human immunodeficiency virus, Parkinson’s disease, and many more [57,58].

### 3.1. Nanoparticles and Drug-Delivery

Nanoparticles used for medical applications are multifunctional, and the drug-delivery function is undoubtedly the most frequently used. The utilization of nano-carriers during cancer therapy alleviates the non-specific accumulation and side effects of anticancer drugs into normal cells, thus, offering better tumor targeting and enhancement of therapeutic efficiency. The increasing utilization of nanoparticles in drug-delivery is justified by the inadequacy of conventional therapeutic agents alone to target affected tumor tissues and effectively treat the condition. Currently used nano-drug delivery systems include micelles, dendrimers, liposomes nanotubes, and various other carriers (polymeric-, solid lipid-, viral-, gold-, and magnetic-carriers) [59,60]. Such nanoparticles-specific delivery could be achieved by two routes known as passive and active targeting [61]. The first is more common and based on two distinctive characteristics of the tumor microenvironment, namely, the enhanced permeability and retention (EPR) effect and acidic conditions. The EPR effect is triggered by the high metabolic capacities of tumor cells and ever-developing neovascularization, which is often porous with gap junctions between endothelial cells. It is through these breaches that passive targeting becomes possible and nano-carrier systems selectively accumulate in tumor cells. In order to meet the high energy demand of ever-proliferating cells, the high oxygen demand causes the activation of the glycolytic pathway, resulting in an acidic extracellular tumor environment. Once incorporated in tumor cells, which are in acidic surroundings, nano-carrier systems like liposomes disintegrate and release therapeutic agents. The active routes aim to reinforce selective targeting and overcome the problems occurring during passive targeting such as the mucosal barrier and non-specific drug-delivery. Thus, active nano-carrier systems are made even more specific to tumor cells through conjugation to biomolecules including ligands and antibodies [59,62,63,64].

### 3.2. Essential Bonding in Carrier-Systems

The usage of targeted anticancer agents aims to improve the therapeutic outcomes and release the active form of the drug at its lowermost dose with minimal activity loss and side effects. The prolonged accumulation of drugs into cancer cells and their subsequent improved therapeutic index are counted among the direct benefits of targeted anticancer therapy. Thus, the nature of bioconjugation and bonds between the carriers and drugs in one hand, and between carriers-drug systems and cancer cells on the other hand, are of crucial importance [48]. Both covalent and non-covalent interactions are employed to assemble an effective anticancer drug-delivery complex. Amide bond formation on the surface of nanocarriers is achieved via a stable chemical reaction. Minimal activity loss, high stability and dispersion of resulting complexes in an aqueous environment, prolonged biodistribution, and large accumulation in cancer cells, as well as enhanced therapeutic outcomes, can be obtained using modified targeted delivery systems [48,65,66].

Thioester bond formation between nanocarriers and ligands yields high selectivity of delivery systems and prolonged biodistribution. Such formation requires adequate chemical reactions, with potential molecular rearrangement and disulfide bond formation [67,68]. Disulfide bonds could occur following conjugation of ligands and nanocarriers, and these delivery systems may well possess a stronger affinity for cancer cells [67,69]. Hydroxyl groups attached to ligands, render them able to interact with hydrazide groups of nanocarriers, forming acetyl-hydrazine bonds. Such modifications bring control and stability to the whole system and enable it to last in blood and a high immunological environment [48]. Another way to increase the affinity to cancer cells is to modify ligands by the Diels-Alder reaction to yield bicyclic products. The same reaction can also be used to bond ligands to nanocarriers [67,68,69,70]. On the other hand, non-covalent interactions are fragile linkages with the delivery systems and simply broken during treatment. The increasing possibility of ending up with side-products after the modification of delivery systems is becoming a quality and safety issue. Often neglected after assembling processes, purification steps are required and the click chemistry may provide purification options for such systems before their use in targeted cancer therapy [71].

Despite significant progress in cancer management observed over the past years, the curative efficiency has not improved concurrently. This may be due to a minor CSCs population with distinctive abilities, responsible for treatment resistance, metastatic growth, and tumor recurrence [72]. Thus, the existence of CSCs is a major obstacle for cancer treatment and more efforts have to be consented to develop cancer therapies that concurrently target CSCs. Certainly, a possible and promising route to achieve such an objective is to use nanomedicines to increase CSCs sensitivity and the efficacy of anti-CSCs treatments [71]. The role of CSCs in self-renewal, proliferation, tumor progression, drug resistance, recurrence, and metastasis in many neoplastic conditions could be minimized by using nanoparticles for better delivery of anti-cancer agents but also targeting CSCs, thus achieving prevention of drug-resistance as well as cancer recurrence. Besides the delivery role, nanomaterials improve the stability and increase the bioavailability of anti-CSCs agents, decrease drug-resistance ability of CSCs, and reduce side effects on normal stem cells [73,74]. Nano-carriers used in drug-delivery systems are highly capable of penetrating the CSC niche and enhancing the therapeutic efficiency by up to 100-fold against drug-resistant lymphoma, breast and prostate cancer in comparison with free drugs. Nano-carriers do so by altering pathways involved in self-renewal and differentiation of CSCs, CSC proliferation as well as regulation of metabolic activities of drug-resistant cells and drug-efflux transporters [19,73,75,76,77]. By using nano-carriers in therapy, it is possible to simultaneously kill cancer cells and eradicate drug-resistant CSCs both in vitro and in vivo [78].

## 4. Nanoparticles in Combined Therapy: Health Promoter or Health Hazards

Similarly to other noble metals, gold nanoparticles (GNPs) have outstanding potential in enhancing the efficacy of cancer therapy, due to their ability to exhibit exclusive physiochemical and optical features, particularly their plasmonic properties. Thus, they are utilized in targeted drug and gene therapy; and their roles include the incorporation of PSs in multi-component delivery systems and the facilitation of PS’s transcytosis across epithelial and endothelial barriers. PDT is emerging as a therapeutic solution for cancer and other diseases. However, several PSs used in PDT are highly hydrophobic and require carrier systems to enhance cellular targeting and uptake by the cell. GNPs can enhance not only the cellular uptake of PSs but also the singlet oxygen generation and efficiency of PDT [79,80]. The usage of GNPs and subsequent localized surface plasmon have been recognized as reasons for the enhancement and efficiency of combined therapeutic approaches. GNPs-mediated cancer therapy had offered great specificity and minimal side-effects coupled with effective cancer destruction both in vitro and in vivo [81,82,83].

Although the use of GNPs and other nanomaterials have shown beneficial properties for the management of cancers among other conditions, clarification on a number of safety issues is still needed. First, the exact amount of nanomaterials in targeted diseased areas should be determined prior to the proper determination and application of other parameters such as irradiation of the targeted areas during combination therapies. The irradiation of deep cancer tissues and monitoring of the therapy still require much technological development to assess the targeted cellular sites [84]. Contrary to PSs, nanoparticles are toxic to cells to some extent [85,86], as shown in Table 1. The lack of proper safety and evaluation tools for nanoparticles during cancer therapy constitutes a major setback. The use of immunomodulatory and enhanced surface modification approaches could help to alleviate the potentially hazardous effects of certain nanoparticles such as the metal nanoparticles [54,71,73]. Due to their relatively small sizes, nanoparticles are easily taken up by cells and could lead to an exponential increase, extended accumulation, cell damage, and, eventually, nano-pollution in the hosts. The multiple active agents in nanoparticles blends and possible diverse biological behaviors make it difficult when undergoing standard drug analysis, thus constituting a hurdle for advanced medical applications. Nanoparticles are mostly used as delivery agents rather than actual therapeutic agents and there is very little research on the effect of nanoparticles post-delivery. Any potentially good therapeutic approach using such materials for delivery purposes should provide a clear indication of their removal from biological systems. The absence of such a provision renders the use of nanoparticles as ambiguous in any cancer therapeutic approach, especially for clinical trials and further applications. Small sized nanomaterials (1–30 nm) should readily be cleared via the kidney route, while larger ones are taken up by Kupffer cells and macrophages for excretion by the liver or spleen [59]. Early in vitro evidence showed that nanomaterials remained in cellular compartments for up to three weeks before being cleared by exocytosis [71]. The determination of the resulting impact of such delayed clearance would elucidate the influence of this technology in the battle against cancer.

## 5. Conclusions

The success observed with the use of conventional therapeutic approaches to cancer is mostly due to early screening and diagnosis. Late detection and increasing prevalence of drug resistant cancers require further research and treatment development. Nanomedicine used in conjunction with other upgraded therapies would be a better alternative for cancer eradication, providing that all the setbacks and clarifications are dealt with. The preeminence of nanomedicine in cancer therapy over current treatment options will continue to increase and possibly result in the effective eradication of drug-resistant cancers. Nanoparticles-based medical applications have already shown tremendous benefits in fighting various conditions. In cancer therapy, this new technology offers the potential to alleviate the persisting problems of cancer recurrence and drug-resistance. GNPs stand as distinguished intracellular targeting carriers due to size-tailoring, multiple surface-functionalities, and exceptional light-dependent features. The fantasy of the prolonged effects of nanoparticles in desired cells, tissues, and organs creates a safety issue, which is the major limitation; therefore, rendering the technology unattractive for some advanced applications. Purification of delivery systems post-modification, as well as post-delivery clearance, seem to be ignored. Whether nanomedicine is a boon or bane for cancer therapy, can only be addressed after further investigation and expansion of the technology. However, the feasibility of this technology leaves plenty of room for improvement and modification to achieve a strong affinity to cancer cells, render delivery systems able to endure immunologic attacks and yield even better therapeutic outcomes. Nanomedicine is the future of cancer therapy as it offers the prospect to overcome the current limitations, which require detailed insights for specific targeting approaches, improved cellular uptake for all tumors, including CSC populations, and an optimal remedy with enhanced efficiency.

## Figures and Tables

**Table 1:** Potential applications and toxic effects of nanomaterials.

Types	Name	Main Applications	Toxicity and Affected Structures	References
Metallic	Aluminum oxide	Fuel cells, polymers, paints, coatings, textiles, biomaterials	Cell viability, mitochondrial functions, oxidative stress, protein expression, genotoxicity	[87,88]
Metallic	Gold, easily functionalized	Drug-carriers, contrast agents	Relatively safe, non-toxic spherical core	[50,89]
Metallic	Copper oxide	Antibacterial agents, semiconductors, heat transfer fluids, contraceptive devices	Cell membrane integrity, oxidative stress, liver, kidney, spleen, genotoxicity	[90,91,92]
Metallic	Silver	Antibacterial agents, wide range of commercial products, wound dressing, coating surgical instruments, prostheses	Cell viability, cell membrane integrity, oxidative stress, kidney, liver, lung, genotoxicity	[93,94,95]
Metallic	Zinc oxide	Wave filters, UV (Ultra-Violet) detectors, gas sensors, sunscreen, body care products	Cell viability, cell membrane integrity, mitochondrial functions, oxidative stress, liver, genotoxicity	[96,97,98]
Metallic	Iron oxide	Drug-carriers, diagnostic agents	Cell viability, mitochondrial functions, oxidative stress, brain, liver, lung, genotoxicity	[99,100,101]
Metallic	Titanium oxide	Pigment and coloring agents	Oxidative stress, immune function, lung, liver, kidney, spleen, genotoxicity	[102,103,104]
Non-Metallic	Carbon-based and Fullerenes	carbon nanotubes	Cell viability, cell membrane integrity, liver, kidney, bone, spleen, genotoxicity	[105,106,107,108]
Non-Metallic	Silica, easy functionalized	Drug-carriers (easy functionalized)	Oxidative stress, cell membrane integrity, mitochondrial functions, genotoxicity	[109,110,111]
Non-Metallic	Polymers (biodegradable)	Drug-carriers	Relatively safe, non-toxic, non-immunologic, non-inflammatory, least toxicity	[112]
